# Nontuberculous Mycobacteria, Zambia

**DOI:** 10.3201/eid1502.080006

**Published:** 2009-02

**Authors:** Patricia C.A.M. Buijtels, Marianne A.B. van der Sande, Cas S. de Graaff, Shelagh Parkinson, Henri A. Verbrugh, Pieter L.C. Petit, Dick van Soolingen

**Affiliations:** Medical Centre Rijnmond-Zuid, Rotterdam, the Netherlands (P.C.A.M. Buijtels); University Medical Center Rotterdam, Rotterdam (P.C.A.M. Buijtels, H.A. Verbrugh); National Institute of Public Health and the Environment, Bilthoven, the Netherlands (M.A.B. van der Sande, D. van Soolingen); Medical Centre Alkmaar, Alkmaar, the Netherlands (C.S. de Graaff); St. Francis Hospital, Katete, Zambia (S. Parkinson); Vlietland Hospital, Schiedam, the Netherlands (P.L.C. Petit)

**Keywords:** Tuberculosis, nontuberculous mycobacteria, clinical relevance, diagnosis, Africa, Zambia, research

## Abstract

Clinical relevance of nontuberculous mycobacteria (NTM) isolated from 180 chronically ill patients and 385 healthy controls in Zambia was evaluated to examine the contribution of these isolates to tuberculosis (TB)–like disease. The proportion of NTM-positive sputum samples was significantly higher in the patient group than in controls; 11% and 6%, respectively (p<0.05). NTM-associated lung disease was diagnosed for 1 patient, and a probable diagnosis was made for 3 patients. NTM-positive patients and controls were more likely to report vomiting and diarrhea and were more frequently underweight than the NTM-negative patients and controls. Chest radiographs of NTM-positive patients showed deviations consistent with TB more frequently than those of controls. The most frequently isolated NTM was *Mycobacterium avium* complex. Multiple, not previously identified mycobacteria (55 of 171 NTM) were isolated from both groups. NTM probably play an important role in the etiology of TB-like diseases in Zambia.

## Abstract 2

One-sentence summary for table of contents: These organisms play an underestimated role in tuberculosis-like diseases in this country.

Tuberculosis (TB) is a problem of enormous dimensions in Africa, and *Mycobacterium tuberculosis* is the most important causative agent. However, in industrialized countries, nontuberculous mycobacteria (NTM) also play a key role in etiology of TB-like syndromes. In Africa, the contribution of NTM to such disease has been examined on a small scale only (*1**–**6*).

Zambia is a country with historically high prevalence rates of TB. Patients with acid-fast bacilli (AFB)–positive sputum, or those with chest radiographic findings suggestive of active TB, who do not respond to general antimicrobial drugs, are generally presumed to have pulmonary TB. In general, these patients are treated empirically for 6 months with a combination of drugs recommended by the World Health Organization. However, several TB-like syndromes in Africa could be caused by NTM. Thus, inconclusive diagnosis of pulmonary TB would lead to overdiagnosis of TB and in some cases, to inappropriate treatment for NTM infections.

When NTM are isolated from a usually sterile site (e.g., blood, bone marrow, lymph nodes, synovial fluid), diagnosis of true disease is generally straightforward. However, when NTM are isolated from nonsterile sources, such as sputum or bronchoalveolar lavage samples, the diagnosis is less definitive, especially when the colony numbers are low or NTM are isolated from only 1 cultured specimen. Therefore, it is a challenge to differentiate true NTM lung disease from contamination and colonization. Thus, finding AFB by microscopy of respiratory specimens or by culture may pose a diagnostic problem for the clinician. In 1997, the American Thoracic Society (ATS), published useful criteria for determining the clinical relevance of NTM isolates (*7*). In 2007, the ATS guidelines were revised to include more lenient diagnostic criteria (*8*). Clinical criteria include a symptomatic patient with pulmonary symptoms, nodular or cavitary opacities on chest radiographs, or a high-resolution computed tomography scan that shows multifocal bronchiectasis with multiple small nodules. In addition, the microbiologic criteria comprise positive culture results from >2 separate sputum samples, or positive culture results from >1 bronchial wash or lavage, or a lung biopsy specimen with mycobacterial histopathologic features (granulomatous inflammation or AFB) and positive culture for NTM, or lung biopsy specimen showing mycobacterial histopathologic features and >1 sputum or bronchial washing culture positive for NTM.

In a pilot study performed in 3 hospitals in Zambia in 2001, high rates of NTM culture–positive sputum samples were obtained (P.C.A.M. Buijtels et al., unpub. data). Therefore, we studied the clinical relevance of and risk factors for isolation of NTM from HIV-positive and HIV-negative patients with chronic productive cough and from randomly selected community controls in Zambia.

## Materials and Methods

The study was conducted in St. Francis Hospital in the district of Katete in Zambia from August 2002 through March 2003. Informed consent was obtained from all patients and controls before enrollment. The study was reviewed and approved by the research ethics committee of the University of Zambia, the Central Board of Health, and the Ministry of Health in Zambia.

The study population was composed of adults (>15 years of age) with chronic (defined as >2 weeks) signs and symptoms and a productive cough who were admitted to the department of internal medicine at the hospital. Most (96%) of the included patients had respiratory tract symptoms. The other 4% of the patients had skin infections/abscesses or lymphadenopathy.

For each eligible patient who consented to participate in the study, 2 healthy community controls were recruited randomly from the neighboring community. These controls were not matched for age or other characteristics of the patients. Nested within this case–control study, the characteristics of NTM-positive and NTM-negative persons were analyzed separately.

At the time of enrollment, patients and controls were interviewed in their own language, and their medical records were reviewed by using a standard form. A detailed physical examination was conducted. Chest radiographs were evaluated in a blinded manner in the Netherlands without any additional clinical information. Radiographs were scored for mediastinal adenopathy, cavitation, pleural and pericardial fluid, miliary pathologic changes, alveolar infiltration, interstitial pathologic changes, other lung pathologic changes, or no pathologic changes. Results of the scoring system were chest radiographs with no pathologic changes, pathologic changes not suggestive of TB, and pathologic changes consistent with TB. Over 3 consecutive days, sputum was collected from patients with a productive cough. Controls were asked to gargle with normal saline if they could not produce sputum. The first 2 sputum samples or gargle specimens were cultured for mycobacteria, and a third sample was stored at –20°C until used.

### Laboratory Methods

Sputum or gargle specimens were divided into 2 equal parts: half was decontaminated with *N*-acetyl-l-cysteine–NaOH and half was decontaminated by using 6% sulfuric acid to compare these decontamination procedures for culture of mycobacteria (*9*). Specimens were cultured in Mycobacteria Growth Indicator Tubes (Becton Dickinson Microbiology Systems, Cockeysville, MD, USA) according to the manufacturer’s instructions and guidelines reported by Master (*10*). *Mycobacterium* isolates were identified by using the Accuprobe culture confirmation test for the *M. tuberculosis* complex (Accuprobe; bioMérieux, Marcy l’Etoile, France) or by 16S rRNA gene sequencing (*11*). Serologic testing for HIV was performed by using a qualitative immunoassay (Determine HIV-1/2; Abbott Laboratories, Abbott Park, IL, USA) and the Vidas HIV DUO assay (bioMérieux).

### Data Analysis

Data were entered into SPSS version 6 software (SPSS Inc., Chicago, IL, USA) and analyzed by using STATA version 8.0 (StataCorp., College Station, TX, USA). Student *t*-tests were used to assess different means between groups; proportions were compared by using χ^2^ tests. Univariate odds ratios with 95% confidence intervals were calculated to assess associations of potential risk factors for NTM positivity. A stepwise backward regression approach was used for multivariate analysis.

Body mass index (BMI) was calculated as weight in kilograms divided by squared height in meters. Underweight was defined as a BMI <18.

### Clinical Diagnosis of NTM Lung Disease

NTM lung disease was diagnosed if patients had respiratory symptoms, abnormal chest radiographic results suggestive of *Mycobacterium* infection, and 2 positive sputum cultures with the same NTM. Patients or controls with positive NTM cultures who did not meet these criteria were considered colonized.

## Results

From August 2002 through February 2003, 180 patients and 385 controls were enrolled in the study. Two sputum samples were cultured from 154 patients and 383 controls, and from 1 sample of the remaining participants. The median age of the patients and controls was 35 years (range 16–80 years) and 30 years (range 15–78 years), respectively. Female participants represented 55% (99/180) of the patient group and 69% (265/385) of the control group. A total of 128 (71%) of the patients were HIV positive, and 87 (23%) of the controls were HIV positive. Statistically significant differences in age, sex, and HIV status were observed between patients and controls.

### Culture Results for Patients

Of 180 patients, 60 (33%) had only *M*. *tuberculosis* isolates in their sputum samples, 12 (7%) had *M*. *tuberculosis* and NTM, and 19 (11%) had only NTM (Table 1). Microscopic results of Ziehl-Neelsen–stained smears of sputum samples were positive in 46 (67%) of 69 patients with *M*. *tuberculosis* isolates and in 1 patient with an NTM-positive culture.

**Table 1:** Culture results for 180 hospitalized chronically ill patients and 385 controls, Zambia, August 2002–March 2003*

Results	Patients	Controls
Culture exclusively *Mycobacterium tuberculosis*, no. (%)	60 (33)	2 (0.5)
Culture *M. tuberculosis* and NTM, no. (%)	12 (7)	1 (0.3)
Culture exclusively NTM,† no. (%)	19 (11)	61 (16)
Culture NTM,‡ no. (%)	31 (17)	62 (16)
2 sputum or gargle samples cultured	154	383
2 sputum or gargle samples cultured from NTM-positive person	29 of 31 NTM-positive patients	62 of 62 NTM-positive controls
1 NTM-positive culture in NTM-positive person with 2 samples cultured	22 of 29 NTM-positive patients	61 of 62 NTM-positive controls
1 NTM-positive cultures in NTM-positive person with 2 samples cultured	4 of 29 NTM-positive patients	1 of 62 NTM-positive controls
2 NTM-positive cultures in persons with 2 samples cultured§	4 of 154 patients with 2 samples cultured	1 of 383 controls with 2 samples cultured

*NTM, nontuberculous mycobacteria. †Proportion of patients with exclusively NTM was comparable with controls (p = 0.2). ‡NTM isolated with or without *M*. *tuberculosis*. §Significantly more patients than controls had 2 sputum or gargle cultures positive for NTM (p<0.05).

Of the 31 NTM-positive patients, 29 had 2 consecutive sputum samples subjected to culture. In 22 of the 29 patients, only 1 of 2 cultures was positive. Four NTM-positive patients had 2 positive NTM cultures; 1 of these patients had pulmonary disease. *M*. *intracellulare* was isolated twice from 2 of these 4 patients, *M*. *avium* was isolated from both samples from 1 patient, and NTM of 2 species and *M*. *tuberculosis* was isolated from 1 patient. One of 2 sputum cultures from 3 other NTM-positive patients contained mycobacteria that could not be identified. Two of these 3 patients had *M*. *intracellulare* in 1 sputum sample; *M*. *porcinum* was isolated from 1 patient.

### Case Reports for Patients with Suspected NTM Disease

Characteristics of the 4 patients with NTM isolates in both sputum samples and of the 3 NTM-positive patients from whom 1 of the 2 sputum samples contained mycobacteria that could not be identified are shown in Table 2. For 3 patients (1, 2, and 4), NTM-associated disease was suspected because of the combination of symptoms, positive cultures, and pathologic changes consistent with TB seen on chest radiographs. However, only 1 patient (patient 4) fulfilled the ATS criteria for NTM lung disease. *M*. *intracellulare* was isolated from all 3 of these patients.

**Table 2:** Data for 4 patients with NTM in 2 consecutive sputum samples and 3 NTM-positive patients from whom 1 of 2 sputum samples contained mycobacteria that could not be identified, Zambia, August 2002–March 2003*

Patient no.	Isolate from first sputum sample	Isolate from second sputum sample	Ziehl-Neelsen staining	Sex	Age, y	HIV status	Temp, °C	Duration, wk	Chest radiograph result	Died	BMI
1†	*Mycobacterium intracellulare*	AFB not identified	1+	M	55	+	34.6	17	Suspected TB	Yes	17
2†	AFB not identified	*M. intracellulare*	–	F	45	+	36.1	487	Suspected TB	No	20
3	*M. intracellulare*	*M. intracellulare*	–	M	43	+	36.3	3	No pathologic changes	No	19
4†	*M. intracellulare*	*M. intracellulare*	–	M	32	+	36.8	17	Suspected TB	Yes	15
5	AFB not identified	*M. porcinum*	–	F	50	–	36.6	8	No suspected TB	No	21
6	*M. avium*	*M. avium*	–	M	33	+	37.4	17	No radiograph	No	16
7	*M. avium*	*M. peregrinum*, *M. tuberculosis*	–	F	24	+	35.0	13	No pathologic changes	Yes	NK

*NTM, nontuberculous mycobacteria; Temp, temperature at time of enrollment; Duration, duration of symptoms from time of coming to the hospital to enrollment in the study; BMI, body mass index; AFB, acid-fast bacilli; TB, tuberculosis; NK, not known. All patients had respiratory symptoms. †Patients assumed to have pulmonary NTM disease.

The first patient (patient 4) was a 32-year-old HIV-positive man who reported having had a productive cough with hemoptysis for 17 weeks. He also was vomiting and had diarrhea. His BMI was 15. He had been treated for TB. Results of a chest radiograph were consistent with TB and showed alveolar infiltration and interstitial pathologic changes. His condition did improve after treatment with antimicrobial drugs (chloramphenicol and tetracycline), and he was again given treatment for TB. Mycobacteria were cultured from 2 sputum samples and identified as *M*. *intracellulare*. The patient died 5 weeks later.

The second patient (patient 1), who had *M*. *intracellulare* pulmonary disease, was a 55-year-old HIV-positive man. He was admitted because of a productive cough with hemoptysis for 17 weeks. Diarrhea was also reported; his BMI was 17. He had been receiving treatment for TB for 4 months. Sputum obtained before treatment was AFB negative. Radiographic investigation of the chest showed cavities and alveolar consolidation. No improvement was seen after he was treated with antimicrobial drugs (chloramphenicol, amoxicillin, gentamicin, and metronidazole). Sputum was examined again and was smear positive for AFB. The first sputum culture showed *M*. *intracellulare*. In the second sputum culture, the isolated mycobacteria could not be identified because of logistic reasons. The patient died 3 weeks later.

The third patient (patient 2), who had *M*. *intracellulare* pulmonary disease, was a 45-year-old HIV-positive woman who had had respiratory signs and symptoms for >1 year. She was known to have asthma. Physical examination found enlarged submandibular, supraclavicular, and axillary lymph nodes. Her BMI was 20. Alveolar infiltration was seen on a chest radiograph. Treatment with chloramphenicol was started. Culture of the first sputum sample showed mycobacteria that could not be identified; the second sputum showed *M*. *intracellulare*. Three days after admission, the patient was taken home by her family and was lost to follow-up.

### Culture Results for Controls

*M*. *tuberculosis* was cultured from sputum or gargle specimens from 3 (0.8%) of the 385 controls; in 1 of these 3 controls, NTM and *M*. *tuberculosis* were isolated (Table 1). In 61 (16%) controls, only NTM were isolated; this number was comparable with the proportion of patients among whom only NTM were isolated (11%; p = 0.2). In 61 of 62 NTM-positive controls, only 1 sputum or gargle specimen was culture positive for NTM. In 1 control, who was HIV negative, 2 mycobacteria, *M*. *porcinum* and an unknown *Mycobacterium* sp., were isolated. No chest radiographs suggestive of TB were observed for any of the controls.

From 383 controls, 2 sputum or gargle samples were cultured for *Mycobacterium* spp. Significantly fewer controls (1/383) than patients (4/154) had 2 sputum or gargle cultures positive for NTM (p<0.05).

### *Mycobacterium* spp. Isolated

To compare the influence of the decontamination method on the yield of mycobacteria, we divided sputum or gargle samples from all patients and controls into 2 equal parts before decontamination (*9*). A total of 635 sputum samples were cultured from 180 patients, and 1,532 sputum or gargle samples were cultured from 385 controls. The results of the cultures are shown in Table 3. The number of NTM (72) isolated from 635 sputum samples of patients was significantly higher than the number of NTM (99) isolated from 1,532 sputum or gargle samples from controls (11% and 6%, respectively, (p<0.001).

**Table 3:** Results from cultures of sputum samples taken from hospitalized chronically ill patients and controls, Zambia, August 2002–March 2003

Result	Patients, no. (%)	Controls, no. (%)
Negative	362 (57)	1,428 (93)
*Mycobacterium tuberculosis*	201 (32)	5 (0.3)
*M. avium* complex	15 (2)	5 (0.3)
*M. intracellulare*	12 (2)	0
*M. avium*	3 (0.5)	5 (0.3)
*M. gordonae*	4 (0.6)	0
*M. peregrinum*	2 (0.3)	4 (0.3)
*M. goodie*	1 (0.2)	4 (0.3)
*M. porcinum*	1 (0.2)	3 (0.2)
*M. lentiflavum*	1 (0.2)	0
Unknown *Mycobacterium* spp.	13 (2)	42 (3)
Other *Mycobacterium* spp.*	3 (0.5)	7 (0.5)
Unidentified acid-fact bacilli	32 (5)	34 (2)
Total no. sputum samples	635	1,532

*Other *Mycobacterium* spp. in patients were *M. fortuitum*, *M. neoaurum*, and *M. simiae*. Other *Mycobacterium* spp. in controls were *M. fortuitum*, *M. asiaticum*, *M. aurum*, and *M. conspicuum*.

Mycobacteria were isolated from 273 (43%) of 635 sputum samples from patients; *M*. *tuberculosis* isolates were found in 201 (74%) of the 273 positive sputum specimens, and NTM were found in 72 (26%). The most frequently isolated NTM was *M*. *intracellulare*, which was found in 12 specimens.

Mycobacteria were isolated from 104 (6.8%) of 1,532 sputum or gargle samples cultured from the controls. *M*. *tuberculosis* was found in 5 (4.8%) of the 104 positive cultures, and NTM were found in 99 (95%). The predominant NTM isolated from the controls were *M*. *avium* (5 specimens), *M*. *goodii* (4 specimens), and *M*. *peregrinum* (4 specimens).

A total of 55 (32%) of 171 NTM isolated from patients and controls were not identified because their 16S rDNA sequences were absent in the BLAST (National Center for Biotechnology Information, Bethesda, MD, USA, www.ncbi.nlm.nih.gov) database. These 55 NTM species were closely related to various *Mycobacterium* spp. such as *M*. *intracellulare*, *M*. *malmoense*, *M*. *fortuitum*, *M*. *smegmatis*, and *M*. *terrae*.

### Comparison of Persons with and without NTM in Sputum or Gargle Samples

The 93 patients and controls with NTM-positive cultures had different clinical and radiographic features than the 472 patients and controls without NTM in sputum samples (Table 4). These persons were more likely to report vomiting and diarrhea, were more often underweight (BMI<18), more often had general malaise, and their chest radiographs more frequently showed changes consistent with TB, such as consolidation and interstitial changes. HIV status and presence of *M*. *tuberculosis* in the sputum culture did not differ between the NTM-positive and NTM-negative groups. There were no significant differences between these groups in terms of age (mean 36.7 years vs. 34.8 years; p = 0.2), sex, smoking habits, alcohol use, and previous treatment for TB (Table 5). The percentages of farmers and of persons in both groups who used unboiled milk were comparable. Moreover, NTM-positive persons used tap water more often than NTM-negative persons (p = 0.004). A subgroup analysis, restricted to patients with NTM and patients without NTM in sputum, yielded similar results (data not shown).

**Table 4:** Clinical data for samples obtained from NTM-positive and NTM-negative persons, Zambia, August 2002–March 2003*

Characteristic	NTM-positive samples	NTM-negative samples	p value	All samples
Persons, no. (%)	93 (16.5)	472 (83.5)	–	565
HIV positive, no. (%)	41 (45.6)	174 (38.5)	0.2	215 (39.7)
*Mycobacterium tuberculosis*, no. (%)	13 (14.0)	62 (13.1)	0.8	75 (13.3)
BMI, mean (SD)	20.2 (4.2)	20.8 (3.7)	0.2	20.7 (3.8)
Underweight, no. (%)	26 (29.6)	92 (20.6)	0.06	118 (22.1)
Vomited, no. (%)	8 (8.6)	18 (3.8)	0.04	26 (4.6)
Diarrhea, no. (%)	12 (12.9)	11 (2.3)	<0.001	23 (4.7)
Lymph nodes analyzed, no. (%)	21 (22.8)	146 (30.9)	0.1	167 (29.6)
Chest radiograph compatible with TB but culture negative for *M. tuberculosis*, no. (%)	5 (26.3)	28 (7.1)	0.003	33 (8.0)
Died, no. (%)	9 (9.7)	26 (5.5)	0.1	35 (6.2)

*NTM, nontuberculous mycobacteria; BMI, body mass index; TB, tuberculosis.

**Table 5:** Background characteristics of NTM-positive and NTM-negative persons, Zambia, August 2002–March 2003*

Characteristic	NTM positive, no. (%)	NTM negative, no. (%)	p value	All persons, no. (%)
Persons	93 (16.5)	472 (83.7)	–	565
Female	61 (65.6)	303 (64.2)	0.8	364 (64.4)
Age, y, mean (SD)	36.7 (13.1)	34.8 (14.6)	0.2	35.1 (14.4)
Farmer	48 (51.6)	245 (51.9)	1.0	293 (51.9)
Used tap water	23 (25.0)	62 (13.2)	0.004	85 (15.1)
Used unboiled milk	15 (16.1)	62 (13.1)	0.4	77 (13.6)
Smoker	5 (5.7)	41 (8.9)	0.3	46 (8.4)
Used alcohol	9 (10.3)	51 (11.2)	0.8	60 (11.0)
Hospitalized	31 (33.3)	149 (31.6)	0.7	180 (31.9)
Previously treated for TB	9 (9.7)	28 (5.9)	0.2	37 (6.6)

*NTM, nontuberculous mycobacteria; TB, tuberculosis.

Independent risk factors for NTM culture–positive sputum were determined by using multivariate analysis. Two factors, underweight (BMI<18) and use of tap water, were independently associated with having an NTM-positive sputum culture (Table 6).

**Table 6:** Crude and adjusted risk factors for isolation of NTM from sputum samples, Zambia, August 2002–March 2003*

Factor	Univariate analysis, OR (95% CI)	Multivariate analysis, OR (95% CI)†
Hospitalized	1.1 (0.7–1.7)	NS
Age >25 y	1.3 (0.8–2.5)	NS
Sex (female)	1.1 (0.7–1.7)	NS
Underweight (BMI <18)	1.6 (1.0–2.7)	1.7 (1.0–2.9)
*Mycobacterium tuberculosis* infection	1.1 (0.6–2.0)	NS
Previous treatment for TB	1.7 (0.8–3.7)	NS
HIV positive	1.3 (0.8–2.1)	NS
Used tap water	2.2 (1.3–3.8)	2.0 (1.1–3.5)
Used alcohol	0.9 (0.4–1.9)	NS
Smoker	0.6 (0.2–1.6)	NS
Used unboiled milk	1.3 (0.7–2.3)	NS
Farmer	1.0 (0.6–1.5)	NS
Chest radiograph compatible with TB but culture negative for *M. tuberculosis*	4.7 (1.6–13.9)	NS

*NTM, nontuberculous mycobacteria; OR, odds ratio; CI, confidence interval; NS, not significant; BMI, body mass index; TB, tuberculosis. †Stepwise backward elimination.

## Discussion

The purposes of this study were to compare the prevalence of NTM in sputum between hospitalized chronically ill patients and community controls and to determine the clinical importance of isolation of NTM. The proportions of patients and controls with positive sputum or gargle cultures for NTM were comparable (11% and 15%, respectively). However, the proportion of NTM-positive sputum samples was higher for patients than for controls (11% and 6%, respectively). This finding suggests that persistent NTM are associated with chronic illness in these patients. It is not known whether culture results were influenced by the method of obtaining specimens. A gargle specimen contains flora of the oropharyngeal mucosa, whereas a sputum sample contains flora of the lower airways. In the patient group, more persons were capable of producing sputum, which may have influenced the yield of positive NTM-positive cultures.

NTM lung disease was definitively diagnosed for 1 patient and probable diagnosis was made for 3 patients (nos. 1, 2, and 4; Table 2). *M*. *intracellulare* was isolated from the sputum samples of these HIV-positive patients. These patients had respiratory symptoms, and chest radiographs showed pathologic changes compatible with TB. Unfortunately, in 2 of these patients, 1 of 2 sputum samples with mycobacteria could not be identified because of contamination and reculture problems.

The combination of symptoms, positive cultures, and pathologic changes seen on chest radiographs are characteristics of NTM infection and suggestive of NTM pulmonary disease. However, the ATS criteria valid at the time of the study were not completely fulfilled because only 2 sputum samples were cultured for mycobacteria on consecutive days, instead of the 3 samples recommended. Furthermore, 2 of these patients suspected of having NTM lung disease had been treated for TB. Because these sputum samples were not tested with molecular amplification techniques for multidrug-resistant *M*. *tuberculosis*, the possibility that they had multidrug-resistant TB could not be excluded (*12**,**13*). Conversely, performance of these nucleic acid amplification tests is generally good for clinical respiratory specimens that are AFB smear positive but less so for specimens that contain fewer organisms or are AFB negative. Moreover, because sputum specimens were not cultured on solid medium, it was not possible to count the number of colony-forming units to distinguish colonization and infection from disease.

Many risk factors for NTM have been identified (*14**–**16*). In this study in a setting in Africa, HIV, sex, and age were not risk factors for NTM. The 2 risk factors for a positive NTM culture were being underweight and having consumed tap water. NTM are natural inhabitants of municipal water systems and soil. A biofilm may form in the water distribution system and be a source of replicating NTM (*17*). Consequently, availability of clean tap water may introduce a serious danger, in particular, to immunosuppressed human populations. Therefore, tap water in Zambia should be tested for NTM.

In this study, patients and controls with NTM in sputum or gargle samples more often had symptoms and signs of general malaise, including diarrhea, vomiting, and being underweight, and chest radiographs for these NTM-positive persons more often showed pathologic changes than did those for NTM culture-negative persons. These symptoms and signs may not be specific for NTM infection, but they may reflect the patients’ poor health in general.

Differences in geographic distribution of NTM species have been reported (*16**,**18**,**19*). The most commonly encountered NTM from clinical specimens in industrialized countries are *M*. *avium* complex (MAC) and *M*. *kansasii* (*20**–**24*). Despite limited studies conducted in Africa, the distribution of NTM is not known. In our study, the most commonly isolated NTM in patients and controls was *M*. *avium* complex. However, 32% of NTM found in both groups in Zambia have not been identified on a species level. This study indicates that the distribution of NTM in Africa may differ from that in Europe and the United States. NTM in Africa may have diverged from NTM in industrialized countries. This hypothesis could be tested by extensive DNA sequencing of semiconserved genes such as those for RNA polymerase B and 65-kD heat-shock protein. Unidentified NTM colonize persons in Africa and can cause disease in some instances. The magnitude of this problem, in addition to the problem of TB, is unknown but deserves more attention.

Rates of NTM colonization and disease that have been reported vary in different areas. In North America and Europe, rates of colonization and disease in the general population range from ≈1–15/100,000 persons to 0.1–2/100,000 persons, respectively (*20**–**23**,**25**–**28*). These rates are largely unknown for most countries in Africa. In South Africa, prevalence rates of NTM colonization of 1,400–6,700/100,000 persons have been reported (*29**,**30*). In gold miners in South Africa, rates of infection were 101/100,000 persons for NTM, 66/100,000 persons for *M*. *kansasii*, and 12/100,000 persons for *M*. *scrofulaceum* (*31**,**32*). Although numbers of cases were small, the estimated rate of colonization in our study in the patient population was 9% (14/154) and the rate of disease was ≈2% (3/154). Two sputum or gargle specimens were collected and cultured from 383 controls in our study. NTM were isolated from both specimens for 1 of 61 controls with >1 sample being culture positive for NTM. This control was not suspected of having NTM pulmonary disease. The estimated rate of colonization in the general population on the basis of this result is 16% (61/383).

NTM probably play a role in the etiology of TB-like disease in Zambia. More extended studies, in terms of duration and size, will be needed to determine the true prevalence of NTM infection in Africa.
